# Investigating the Invasion Pattern of the Alien Plant *Solanum elaeagnifolium* Cav. (Silverleaf Nightshade): Environmental and Human-Induced Drivers

**DOI:** 10.3390/plants10040805

**Published:** 2021-04-20

**Authors:** Nikos Krigas, Maria A. Tsiafouli, Georgios Katsoulis, Nefta-Eleftheria Votsi, Mark van Kleunen

**Affiliations:** 1 Institute of Plant Breeding and Genetic Resources, Hellenic Agricultural Organization Demeter, P.O. Box 60458, Thermi, 57001 Thessaloniki, Greece; 2 Department of Ecology, School of Biology, Aristotle University of Thessaloniki, 54124 Thessaloniki, Greece; tsiafoul@bio.auth.gr (M.A.T.); gkatsou.geotopo@gmail.com (G.K.); 3 Institute for Environmental Research & Sustainable Development, National Observatory of Athens, I. Metaxa & Vas. Pavlou, P. Penteli (Lofos Koufou), 15236 Athens, Greece; nvotsi@gmail.com; 4 Ecology, Department of Biology, University of Konstanz, 78464 Konstanz, Germany; mark.vankleunen@uni-konstanz.de; 5 Zhejiang Provincial Key Laboratory of Plant Evolutionary Ecology and Conservation, Taizhou University, Taizhou 318000, China

**Keywords:** climate, soil disturbance, land-use types, road network, agriculture, GIS, non-native, Greece

## Abstract

Invasive alien plant species have impacts on nature conservation, ecosystem services and agricultural production. To identify environmental and human-related drivers of the invasion of *Solanum elaeagnifolium* (Solanaceae)—one of the worst alien invasive plants worldwide—we conducted an extensive drive-by survey across the Greek territory (presence/absence data; all national major multilane highways; 12–25% of the remaining road network; driven 3–5 times during 2000–2020). These data were linked in GIS with (i) physical environmental attributes (elevation, climate, soil properties) and (ii) type and intensity of human-related activities (land uses, settlements and road type). Compared to previous records, our survey showed that the range of *S. elaeagnifolium* increased by 1750% during the last decades, doubling its main distribution centers and reaching higher elevations. Our study revealed that the presence of *S. elaeagnifolium* is associated with (i) higher maximum temperatures and precipitation in summer and low precipitation in winter, as well as with (ii) soil disturbance related to agricultural activities, settlements and road networks, thus facilitating its spread mainly at low altitudes. Our study elucidates the current invasion pattern of *S. elaeagnifolium* and highlights the urgent need for its widespread monitoring, at least in the noninvaded areas in Greece that have been surveyed in this study. Preventative measures and integrative initiatives should be implemented quickly, and urgently incorporated into current agricultural, road network and conservation-management regimes.

## 1. Introduction

Invasive alien plant species are considered to be major contemporary passengers and drivers of ecological change worldwide, with severe negative impacts on agricultural production [1], delivery of ecosystem services [1,2] and nature conservation, decreasing native biodiversity and thus resulting in biotic homogenization [2].

Land use, disturbance and climate have been identified as general driving factors of plant invasions [3,4]. Numerous environmental drivers of invasions can also be perceived as effects of invasions due to the fact that in many cases, no clear and coherent separation can be made between causes and effects of invasions [5]. For instance, it is not known whether land use per se or change of land uses can be considered as the driver of invasion. Similarly, frequency and/or severity of human-induced disturbances can either promote or reduce invaders’ establishment. On the other hand, land use may incorporate numerous different activities such as agriculture, grazing, forest management, industry, urbanization, etc., and each of them may have different impacts on invasive-species performance [5]. However, as there are many different invasive plant species, each with its own requirements, species-specific knowledge is needed regarding the patterns of invasion and the underlying drivers [4,6,7,8]. Such knowledge will allow the prediction of areas that are susceptible to the introduction and establishment of the invasive species, and is essential to prevent the negative consequences of invasive species overall [4,6,9].

*Solanum elaeagnifolium* Cav. (Solanaceae) is one of the worst invasive alien plants in the Mediterranean Basin, and it is invasive worldwide [10], especially in Mediterranean-type climate zones of South Africa and Australia, as well as in subtropical Africa, South Asia and Oceania [11,12], mainly due to its dispersal ability, impacts and resistance to control. Therefore, it is considered as a pest recommended for regulation [11,12]. It is a noxious, deep-rooting weed native to northeastern Mexico and southwestern USA [12], and probably also Argentina [13]. Thessaloniki in Northern Greece was the starting point of *S. elaeagnifolium*’s invasion in the Mediterranean Basin and Europe. It was probably introduced unintentionally from Texas in the 1930s [12,14]. In Greece, it is locally called ‘germanos’ (literally ‘the German’, because people believed that the Germans introduced it; however, locals noticed its emergence as an invasive alien weed only after the Second World War, probably due to extensive disturbance of habitats by construction works and rapid urbanization of the metropolitan Thessaloniki area). *S. elaeagnifolium* often invades in cultivated, abandoned, managed or disturbed lands, pastures, tourist beaches, cities, urbanized areas and roadsides, or even in forested areas. It is often found on variety of soils ranging from sand to clay, and it is characterized by copious production of sexual and asexual propagules associated with a deep-rooting system. It has a strong vegetative regeneration ability, while fluctuating temperatures of 25/15 °C coupled with adequate moisture provide the optimal conditions for its seed germination and growth [12,14]. The propagules of this invasive plant spread into new areas via livestock and their manure, irrigation water, agricultural machinery and vehicles, growing media of ornamental or fodder plants, ballast and bedding used in animal transport [12]. To date this species is associated with a variety of negative impacts on agricultural production, forestry and the environment [11,15], and thus poses a serious threat to all countries around the Mediterranean Basin. Understanding of the drivers of its current invasion pattern is needed to predict which areas are at risk of invasion by this species and to inform management regimes.

In the context of this study, *S. elaeagnifolium* may rather be considered as a passenger, not a driver of ecosystem alterations [5]. Among Mediterranean regions worldwide invaded by *S. elaeagnifolium*, Morocco and especially Greece are the ones that suffer most [12]. The inventory presented herein was initially triggered by the apparent long persistence and unceasing invasion of this perennial alien plant in numerous cities, roadsides and fields in Greece. The systematic inventory presented in this study aimed to document this observation as fully as possible at the national scale for the first time, and anticipated to offer some insight into the possible role of natural (climate, soils/substrates) and human-induced drivers (disturbance, roadsides, land uses) affecting the invasion pattern of *S. elaeagnifolium* in a severely infested country (Greece). To reveal the pattern and drivers of *S. elaeagnifolium* invasion, we made an extensive inventory regarding the occurrence of *S. elaeagnifolium* across the Greek territory, and we also recorded the noninvaded areas for future monitoring. As Greece is characterized by great variety of climatic conditions, geomorphological elements, vegetation types and high biodiversity [16,17], our study is likely to be representative for many other areas in the Mediterranean biomes. Because many invasive plants first spread in settlements and along the roads connecting them, roadside surveys offer a good way to assess distributional patterns of invasive species [18]. Therefore, we recorded *S. elaeagnifolium* presence and absence along the road network (including the adjacent areas beside the roads) connecting all the major cities of Greece and transecting the rural landscapes and many protected areas of the country. We combined the presence/absence data with environmental and human-related drivers in GIS to reveal the underlying factors associated with the distributional pattern of *S. elaeagnifolium.* More specifically, we tested relations of *S. elaegnifolium*’s occurrence with (i) physical attributes such as elevation, climate and soil properties, and (ii) type and intensity of human-related activities (land-use types, settlements, road network) in order improve our understanding about the invasion drivers of this species and to facilitate future management activities.

## 2. Results

### 2.1. Distribution of Solanum Elaeagnifolium in Greece

**Figure 1:**
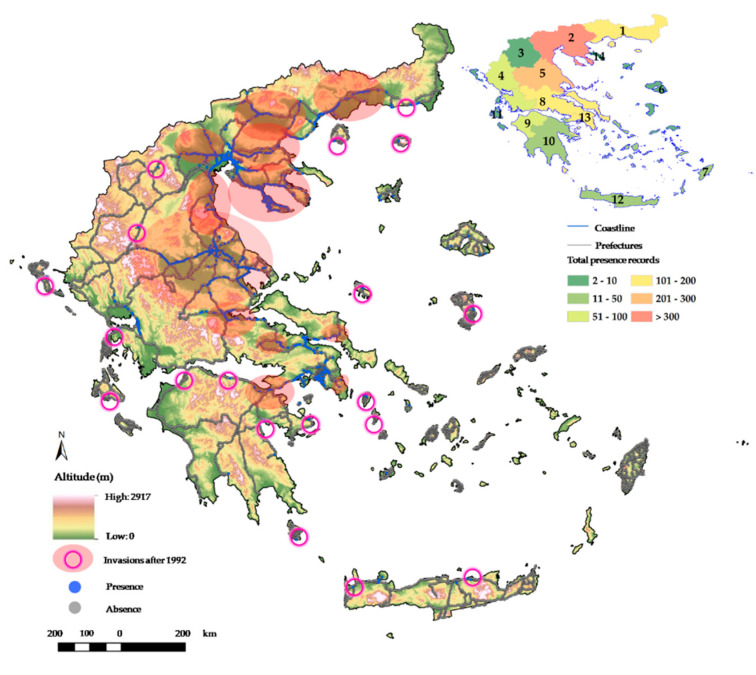
Presence (blue) and absence (grey) records of *Solanum elaeagnifolium* in the studied area of Greece, indicating the invaded and noninvaded areas in Greece with emphasis on invasions that occurred after the last published inventory in 1992 [20], and total presence records of *S. elaeagnifolium* per administrative region (NUTS 2) of Greece. 1: Eastern Macedonia-Thraki; 2: Central Macedonia; 3: Western Macedonia; 4: Epirus; 5: Thessaly; 6: North Aegean Islands; 7: South Aegean Islands; 8: Central Greece; 9: Western Greece; 10: Peloponnese; 11: Ionian Islands; 12: Crete; 13: Attica; 14: Agion Oros (semi-independent monastery region).

This inventory represents the first systematic approach for *S. elaeagnifolium* across the Greek territory. The inventory was made during 15 years and verified during the last five years (see Supplementary S1). The literature survey showed that prior to our drive-by survey, only 84 populations of *S. elaeagnifolium* had been recorded in Greece [19,20,21]. The prior main centers of its distribution in Greece were Central Macedonia, Attica and Western Greece, with only scattered presences elsewhere (see nonshaded presences in Figure 1). Along the road network examined in our drive-by survey, we recorded a total of 1564 cells (out of 15,736 km^2^ cells) that were invaded by *S. elaeagnifolium* (Figure 1). The current main centers of the distribution of *S. elaeagnifolium* in Greece were Central Macedonia and Thessaly with 39.4% and 19.1% of the records, respectively (Figure 1), followed by Central Greece (10.9%), Attica (8.6%), Eastern Macedonia-Thrace (7.4%) and Western Greece (5.4%). Our study indicates that the metropolitan Thessaloniki area and the coastal Chalkidiki regions (Northern Greece) are the foremost distribution centers in Greece. This has been confirmed during the last five years, and our inventory revealed at least three new large distribution centers (and many smaller ones) of *S. elaeagnifolium* that emerged after the 1990s: Thessaly (Larissa, Volos and Trikala), Sterea Hellas (Lamia-Stylida and Chalkida-Schimatari) and Eastern Macedonia-Thrace (Serres and Kavala-Iasmos).

### 2.2. Elevation and Soil Types as Possible Underlying Factors

**Figure 2:**
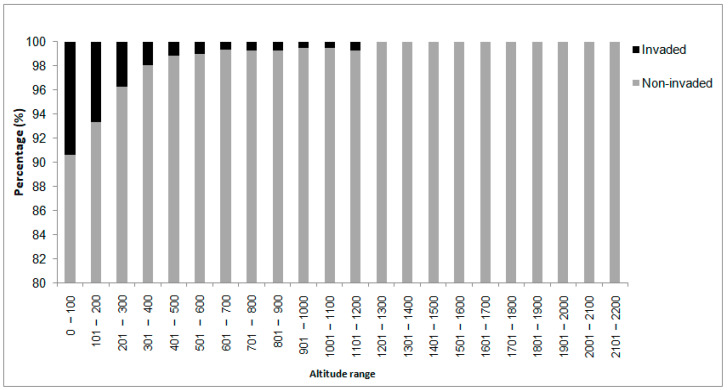
Percentage cover (%) at each class of the altitude range in the noninvaded areas (grey color) and the areas invaded (black) by *Solanum elaeagnifolium* in Greece. Note: the *y*-axis begins at 80%.

The vast majority (60.09%) of *S. elaeagnifolium* records in Greece was found at altitudes from 0–100 m, while 24.58% of the records were from 101–200 m, and 8.53% were from 201–300 m (Table 1). The remaining occurrences were found at altitudes from 301 m up to a maximum of 1200 m. No records of *S. eleagnifolium* were found at surveyed altitudes from 1200 m to 2200 m. Most of the noninvaded areas were also found at low altitudes of 0–100 m (32.84%), 101–200 m (19.46%) or 201–300 m (8.5%), but at lower frequencies than the invaded ones. As shown in Figure 2, the relative percentage of invasion (out of total surveyed) decreased gradually with increase of altitude. This difference in the distribution of *S. elaeagnifolium* across the different classes of altitude was significant according to a chi-square test (Pearson chi-square value: 900.87; asymptotic significance (2-sided) <0.0001).

Values of soil properties for the non-invaded and invaded areas are shown in Table S1 (Supplementary S3) together with results of the chi-square tests performed. The latter showed that the distribution of *S. elaeagnifolium* varied across all studied soil parameters. With regards to the parent material, the majority of invaded areas was found on fluvial clays, silts and loams (46.81%). A similar percentage (44.95%) was found also for the noninvaded areas, showing that this type of parent material is rather common in the surveyed area. A relatively lower percentage of invaded areas (16.69%) compared to the noninvaded ones (23.94%) was found on limestone, resulting in a ratio of 0.7. Similar characteristics for all these soil properties were observed for areas that were not invaded by *S. elaeagnifolium* (Supplementary S3, Table S1), thus indicating its broad tolerance with regard to soil type. Relatively higher percentages for invaded areas compared to noninvaded ones were found for Calcaric Fluvisol (20.29%), Chromic Luvisol (11.46%) and Calcaro-Chromic Vertisol (7.94%), while lower percentages were found for Calcaric Lithosol (13.58). Most of the invaded areas were found on soils with fine texture (63.83%), and in subsoil of medium water availability (44.84%). These percentages were relatively lower than those for the non-invaded areas (67.29% and 36.44%, respectively). The areas invaded in deeper soils (depth to rock) had a relatively higher percentage (33.06%) compared to the noninvaded ones (27.61%), resulting in a ratio of 1.20. On the other hand, there was a relatively lower percentage of invasion in the shallow areas (ratio of invaded/noninvaded areas = 0.72). Finally, most invaded areas were found in soils with high base saturation of the topsoil (61.05%) and low topsoil organic carbon (72.09%). Compared to the noninvaded areas, however, the latter percentage was relatively higher in soils with very low organic carbon (ratio of invaded/noninvaded areas = 0.78).

### 2.3. Climate as Possible Underlying Factor

**Figure 3:**
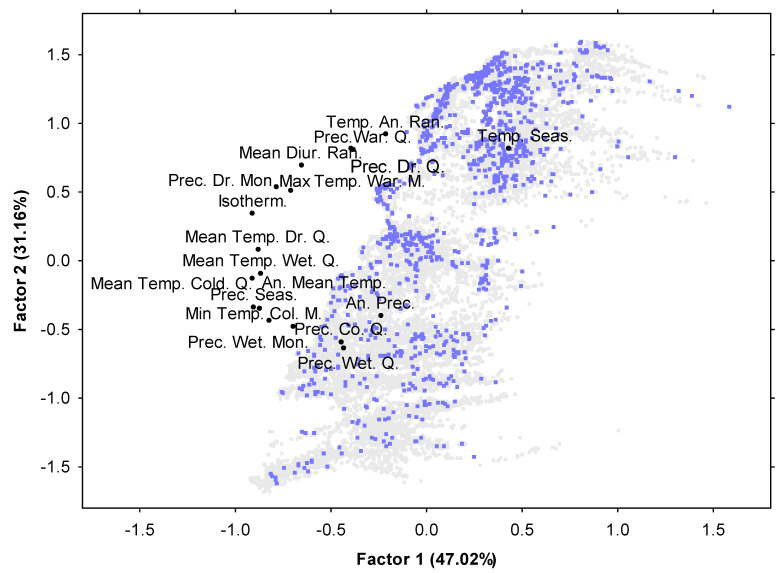
The two first axes of the principal components analysis (PCA) of bioclimatic variables shaping the climate profile in the studied area for the invasion of *Solanum elaeagnifolium* in Greece. Factor 1 and Factor 2 explain 47.02% and 31.16% of variance, respectively. Blue factor scores: invaded areas by *S. elaeagnifolium*; grey factor scores: non-invaded areas by *S. elaeagnifolium*. Factor coordinates: Temp. An. Ran.—Temperature Annual Range; Temp. Seas.—Temperature Seasonality; Prec. War. Q.—Precipitation of Warmest Quarter; Prec. Dr. Q.—Precipitation of Driest Quarter; Mean Diur. Ran.—Mean Diurnal Range; Prec. Dr. Mon.—Precipitation of Driest Month; Max Temp. War. M.—Max Temperature of Warmest Month; Isotherm.—Isothermality; Mean Temp. Dr. Q.—Mean Temperature of Driest Quarter; Mean Temp. Wet. Q.—Mean Temperature of Wettest Quarter; Mean Temp. Cold. Q.—Mean Temperature of Coldest Quarter; An. Mean Temp.—Annual Mean Temperature; Prec. Seas.—Precipitation Seasonality; An. Prec.—Annual Precipitation; Min Temp. Col. M., Min Temperature of Coldest Month; Prec. Co. Q.—Precipitation of Coldest Quarter; Prec. Wet. Mon.—Precipitation of Wettest Month; Prec. Wet. Q.—Precipitation of Wettest Quarter. For analytical presentation of bioclimatic data in the area studied, see Supplementary S3, Table S2.

As shown in the results of the principal components analysis (PCA) in Figure 3, the climatic regime in the areas invaded by *S. elaeagnifolium* largely overlapped with the climatic regime in the noninvaded areas. The most important bioclimatic variables shaping the climate profile in the studied area in Greece along the first PCA axis were: Temperature Seasonality with a positive correlation, and Annual Mean Temperature, Isothermality and Precipitation Seasonality with negative correlations. Along the second PCA axis, the significant variables with positive correlations were related to Temperature Annual Range, Temperature Seasonality, Precipitation of Driest Quarter and Precipitation of Warmest Quarter, while those with negative correlations were Precipitation of Wetter and Coldest Quarters. The PCA Axis 1 and Axis 2 scores differed between the invaded and noninvaded areas according to ANOVA (*p* < 0.001 adjusted by Bonferroni correction), with the invaded areas presenting on average higher PCA1 and lower PCA2 scores than the noninvaded areas. Details regarding the differences in the values of bioclimatic parameters between invaded and noninvaded areas are given in Supplementary S3, Table S2.

### 2.4. Land Uses as Underlying Factor

**Figure 4:**
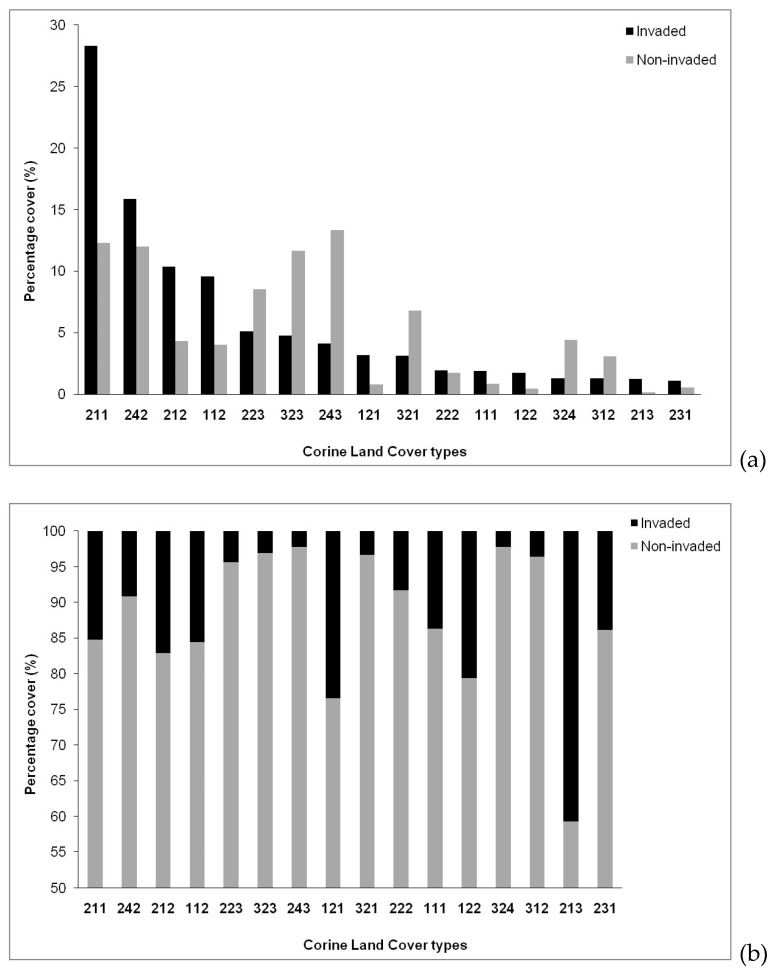
Percentage cover (%) among the various Corine Land Cover types (CLCTs) (**a**) and percentage cover (%) of each Corine Land Cover type (**b**) in the noninvaded areas (grey color) and the invaded areas (black) by *Solanum elaeagnifolium* in Greece. Agricultural CLCTs—211: Non-irrigated arable land; 242: Complex cultivation; 212: Permanently irrigated land; 223: Olive groves; 243: Land principally occupied by agriculture with significant areas of natural vegetation; 222: Fruit trees and berry plantations; 213: Rice fields; 231: Pastures. Forests and semi-natural CLCTs—323: Sclerophyllous vegetation; 321: Natural grassland vegetation association; 324: Transitional woodland shrub; 312: Coniferous forest; 321: Natural grassland vegetation association; 323: Sclerophyllous vegetation; 324: Transitional woodland shrub. Artificial CLCTs—112: Discontinuous urban fabric; 121: Industrial or commercial units’ surfaces; 111: Continuous urban fabric; 122: Road and rail networks and associated land. Land-use types covering less than 1% of the total invaded area are not shown.

Altogether, the land uses of the invaded areas of *S. elaeagnifolium* in Greece were found to belong to 16 different Corine Land Cover types (CLCTs) comprising three categories (agricultural, artificial areas, forests and seminatural areas). Figure 4a (top) shows the percentage cover (%) among the various Corine Land Cover types in the invaded and noninvaded areas. Agricultural land-use types covered altogether over 65% of the distribution area of *S. elaeagnifolium* in Greece, and the most dominant land-use type was 211: Non-irrigated arable land (28.0%), followed by 242: Complex cultivation (16.0%) and 212: Permanently irrigated land (11.0%). Artificial land-use types (mostly 112: Discontinuous urban fabric) covered in total 16.3%, while natural/seminatural land-use types (mainly 323: Sclerophyllous vegetation) covered in total 11.3% of the invaded area. In the noninvaded areas, the CLCTs 211: Non-irrigated arable land, 112: Discontinuous urban fabric and 212: Permanently irrigated land had a much lower percentage contribution (12.0%, 4.3% and 4%, respectively). On the contrary, the CLCTs 243: Land principally occupied by agriculture with significant areas of natural vegetation, 323: Sclerophyllous vegetation, and 321: Natural grassland vegetation association, had higher percentage contributions (13.0%, 12.0% and 6.8%, respectively) compared to the invaded area (see also Figure 4b for percentage cover (%) of each Corine Land Cover type in the invaded and the noninvaded areas. This clear deviation in the distribution of land-use types among the invaded and the noninvaded areas was confirmed by the chi-square tests for most of the above-mentioned CLCTs as follows (the Pearson chi-square value and 2-sided asymptotic significance are given) per CLCTs: 211 (V: 314.078, AS < 0.0001), 212 (V: 280.350, AS < 0.0001), 243 (V: 355.833, AS < 0.001), 112 (V: 163.713, AS < 0.001), 323 (V: 105.053, AS < 0.001), and 321 (V: 65.766, AS < 0.0001).

### 2.5. Road Type as Underlying Factor

In absolute terms, most records of *S. elaeagnifolium* (36.47%) were found next to rural roads (covering 1922 km of this road type), and the fewest records (12.79%) were found along multilane highways (covering 674 km of this road type; Table 2). However, if the surveyed length of the different road types was considered, the multilane highways showed the highest percentage of invasion (73.10%), followed by rural roads (48.33%), old interstate roads (27.81%) and local roads (22.39%).

### 2.6. Distance from Settlements as Underlying Factor

**Figure 5:**
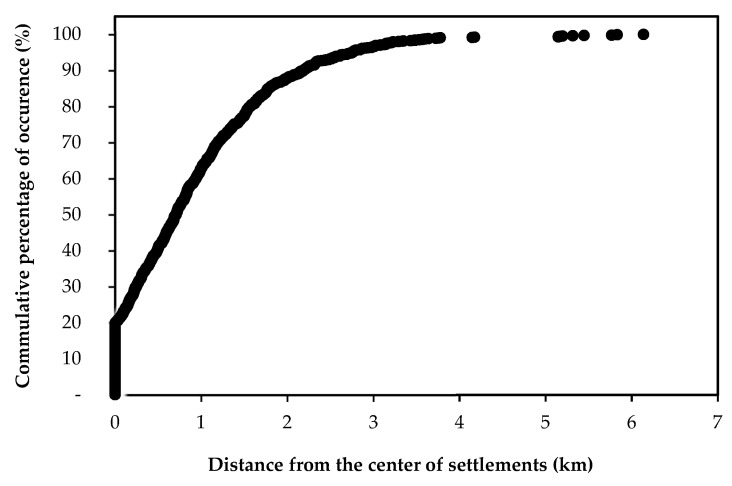
Cumulative occurrence records of *Solanum elaeagnifolium* in relation to the distance from the center of Greek settlements.

All records in the distribution cells of *S. elaeagnifolium* in Greece were found at distances lower than 5 km from the center of settlements (Figure 5). Most of the records of *S. elaegnifolium* are found within settlements (mostly at 1 km from the city centers). From the center of settlements, the presence of *S. elaegnifolium* dramatically decreased within 4 km (Figure 5). It should be noted here that the longest transect of the surface area covered by many capitals of Greek prefectures (e.g., Agios Nikolaos, Alexandoupoli, Corinth, Igoumenitsa, Kalamata, Livadia, Polygyros, Serres, Sparta, Xanthi, etc.) or rural Greek towns and villages (e.g., Almyros, Farsala, Iasmos, Litochoro, Nea Moudania, Stylida, etc.) is often up to 5 km of continuous urban tissue. However, *S. elaeagnifolium* populations may be recorded for a distance up to 18–40 km from big cities like Athens or Thessaloniki (data not shown).

## 3. Discussion

### 3.1. Current Invasion of Solanum Elaeagnifolium in Greece

Our study documented a total of 1564 populations of the invasive alien *S. elaeagnifolium*. We recorded the species in almost all administrative regions of Greece, and this alien plant is still invading new areas across the country (e.g., additional observations in Spetses and Amorgos Islands, Ierapetra area in Crete and Monastery Vatopediou, Agio Oros, not shown in Figure 1). Compared to aggregated data from previous studies [14,19,20,21], we found an alarming 1750% increase of its distribution range in Greece. It is worth mentioning that invasive populations of this alien species have been found even in protected areas of the Natura 2000 network (e.g., GR1220001—LIMNES VOLVI KAI LAGKADA—EVRYTERI PERIOCHI, etc.; Krigas et al. in preparation). Our study thus considerably improves the knowledge regarding its level of invasion in Greece [11,12,22], and illustrates its alarmingly rapid spread. In addition to presence records, we also documented absence records (noninvaded areas) (Figure 1), which could serve as a baseline for future monitoring of the spread of *S. elaeagnifolium* across the Greek territory.

The populations of *S. elaeagnifolium* may outcompete native species (ruderals) of the Mediterranean such as *Cichorium intybus*, *Malva sylvestris*, *Convolvulus arvensis*, *Parietaria judaica* and *Lactuca seriola* [23,24] due to a combination of characteristics such as alternating life strategies (chamaephyte, hemicryptophyte or geophyte) [24], fast occupation of available temporal niches of reduced competition left unexploited [25], long fruiting period (spring till winter in Greece), predominately self-compatible (in native range) or often self-incompatible (in Greece) with berries including >100 seeds [26], rapid growth and copious production of sexual and asexual propagules associated with a deep rooting system [12], strong tillage-induced vegetative propagation, allelopathic effects [27], toxicity to most livestock and absence of enemies in its alien range [12,14].

### 3.2. Elevation and Climate Limitations

Most *S. elaeagnifolium* populations in Greece (95%) are restricted to elevations up to 300 m. In these lowland coastal areas (plains, agricultural lands, urban fabric, road networks), both soil disturbance and propagule pressure are frequent [20,21,22]. However, the spread of alien invasive species to higher altitudes has been facilitated in the last centuries, due to increasing human-induced disturbances and propagule pressure [28,29]. Although we only found three populations of *S. elaeagnifolium* in Greece at higher elevations (900–1100 m), this nevertheless shows that the species can occur at high elevations. Such occurrences seem to be comparable to highland records in its native range [12], thus indicating for the first time the ability of this species to spread into high elevation environments in its non-native range.

In general, alien species are invasive in regions that are climatically similar to their native habitats [9,30,31]. Previous studies on the autoecology of *S. elaeagnifolium* in its native range suggest that it grows in a variety of climatic conditions and that it is well adapted to semiarid conditions [32], while in its alien range in the Southern Hemisphere, it was shown to be adapted to dry winters and wet summers [33]. Although the deep root system enables *S. elaeagnifolium* to endure considerable drought, it seems to prefer places with summer moisture or irrigation regimes [13,22]. We found that *S. elaeagnifolium* is distributed in areas with a relatively high annual and summer precipitation. In their native range, hexaploid cytotypes thrive in wetter areas, whereas tetraploid and diploid cytotypes are found in drier areas [32]. However, it is not known if this applies to the Greek populations.

We found that the climate of the areas invaded by *S. elaeagnifolium* in Greece is a narrower part of the climatic conditions surveyed across the country. Higher values of climatic parameters such as Temperature Annual Range and Seasonality and Maximum Temperature and Precipitation in the Warmest Quarter or Driest Month and lower values of Minimum and Mean Temperature of the Coldest Quarter and Month, as well as Annual Precipitation and Precipitation in the Coldest and Wettest Quarter (or Month) seem to favor its distribution. As diurnally fluctuating temperatures of 25/15 °C coupled with adequate moisture provide the optimal conditions for seed germination and growth of *S. eleaegnifolium* [34], its invasive Greek populations seem to thrive in areas with cold but rather drier winters, and hotter but wetter summers. Preliminary climate-matching models have identified rather similar conditions in other parts of the alien range of *S. elaeagnifolium*, especially in southern Europe, the Mediterranean coastline and south of the Sahara region [11,30]. It has also been suggested that cool and wet summers and high annual rainfall are factors that could prevent the invasion of *S. elaeagnifolium* in certain areas of the Southern Hemisphere such as Australia and South Africa [33]. Our results offer some support for this assumption, since noninvaded areas in Greece seem to have comparatively higher annual and seasonal precipitation values in comparison to the invaded ones (Supplementary S3, Table S2). However, in Europe, it seems that its northern limit of invasion may be more dependent on low winter temperatures and prolonged wet conditions, as established plants are known to be sensitive to frost and waterlogging [35].

### 3.3. Influence of Soil

In its native range, *S. elaeagnifolium* is usually found in sandy, loamy and dry soils but has been recorded in virtually all soil types [13]. We found most Greek populations in fluvial clays, silts and loams, followed by limestone (predominately Calcaric soil types according to FAO) with a fine soil texture, relatively easily available subsoil water capacity and low topsoil organic carbon content. These characteristics seem to favor this deep-rooting weed, but the diversity of soil types this species was found in indicates that the species has a wide edaphic tolerance. The noninvaded sites showed slight deviations in some of the soil properties compared to the invaded sites. However, overall the soil characteristics of the area surveyed are in general likely to be adequate (if not favorable) for *S. elaeagnifolium*, and the species could still spread into currently noninvaded sites. It is known that historical land-use patterns and soil disturbance from previous agricultural and urban land-use changing soil characteristics are important determinants of the current establishment of populations of invasive plants [36]. This indicates that soil functioning may be more influential on invasiveness rather that the soil type. The areas around the main distribution centers of *S. elaeagnifolium* in Greece (Figure 1) have been similarly managed for centuries, and several soil threats have been identified [37]. In any case, the soils in the dominant land-use types of the invaded areas by *S. elaeagnifolium* (i.e., agricultural and urban) are known to be highly disturbed. This, in soil functional terms, usually leads to the suppression of specific biological functions, such as the ability of biological control imposing barriers to invasive species establishment [38,39,40,41].

### 3.4. Type and Intensity of Land Use as Drivers

This study provides evidence that *S. elaeagnifolium* does not spread only along the road network examined but also in the neighboring matrix. We found that in the areas invaded by *S. elaeagnifolium* in Greece, land use is predominantly agricultural or related to urban fabric and road networks. This preference of *S. elaeagnifolium* for human-influenced areas has been also recorded in other studies, both in its native and alien ranges [12,14,22,42].

It is known that cities and settlements act as dispersal centers of alien plants [43]. Indeed, 99% of the *S. elaeagnifolium* records were found within a maximum of 3 km distance from the center of Greek settlements. This suggests that settlements serve as local dispersal centers, and *S. elaegnifolium* populations may further spread into other areas through road verges, which connect the settlements. Most of its populations were found along rural low-traffic roads, something that was expected as this road type is commonplace in Greece [17]. On the other hand, almost 25% of the areas close to the multilane national highways (fully examined throughout Greece) were found to be invaded. It is very possible that high-speed multilane highways and complex road systems produce locally strong turbulence from passing vehicles, which facilitate the effective fruit dispersal of *S. elaeagnifolium* in multiple directions (the round shape of the berry facilitates its rolling due to turbulence from passing vehicles), allowing germination and establishment of new individuals in remote distances from the mother plant. Additionally, management of the verges of high-speed multilane highways is certainly more difficult compared to other road types; however, management of roadside vegetation may induce its further spread through viable root fragments. Moreover, nutrient-rich stormwater run-off from impervious surfaces such as roads may enrich nearby surfaces, thus favoring the vegetative growth and further spread of this species [44].

Road networks and associated land uses seem to be the key factors of the invasion pattern of *S. elaeagnifolium* in Greece. Our analysis revealed some land uses that differed in intensity and type of agricultural management. The first one refers to nonirrigated arable land (28% of the invaded areas in Greece), which is used for crops (e.g., winter wheat) that do not require much water, being rain-fed during the moist season of the year and harvested during the dry one. In fact, the main distribution centers of *S. elaeagnifolium* coincide spatially with the areas of winter wheat cultivation in Greece, as illustrated in [45]. As no irrigation water is used and only limited chemical inputs and fertilizers are used, tillage is the most intensive agricultural practice in such areas. Tillage is known to be one of the most important disturbance factors for the soil system, disrupting the soil structure and affecting soil physical properties [46], soil biodiversity [41] and soil-ecosystem services [38], thus creating easily penetrable soils prone to invasion by alien plants [38,47], and leading to reduced biocontrol efficiency. Tillage is commonly performed after crop harvest (while *S. elaeagnifolium* is in full growth), and the deep-rooted rhizomes of *S. elaeagnifolium* are cut and the still viable fragments are dispersed by tillage. The strong vegetative propagation of *S. elaeagnifolium* from cut root sections renders it almost invincible for farmers [14,22]. Local farmers in the largely invaded areas of Greece (e.g., metropolitan Thessaloniki region) state that ‘*the more you plough your field, the more tillering of S. elaeagnifolium is observed*’ (Krigas, personal communication). They refer to this plant as the ‘*invincible Lernaean Hydra*’, thus alluding to the Greek mythical serpentine water monster that regenerated more heads when severed by the ancient hero Hercules.

Other land-use types found refer to intensively managed agricultural lands (either arable or nonarable) and related road infrastructures (in total, 32% of the invaded areas in Greece). In these areas, there is again the effect of soil disturbance due to tillage, but most importantly the effect of the relatively dense network of roads serving these intensively managed agricultural areas. The species’ fruits may be dispersed by passing vehicles and may float over long distances along rivers, streams (especially during floods) and irrigation systems [12,13]. Although the plants die back in winter, their ripe fruits are retained for long periods on dead branches and may be dispersed by wind or due to turbulence from passing vehicles [13]. Seeds of *S. elaeagnifolium* are also easily and widely dispersed by agricultural machinery, vehicles and tools, as contaminants of hay, alfalfa or ornamental plants, and possibly in the dung of livestock [12]. In this way, all the landscape units formed by the combination of agricultural land and serving road networks represent an excellent environment that offers numerous opportunities for the establishment and invasion of new populations of *S. elaeagnifolium*.

Further land-use types are related to natural/seminatural land uses with extensive agricultural land uses (11% of the invaded area in Greece). The natural land-use types invaded (i.e., grasslands and sclerophyllous vegetation) are among those predicted with high levels of invasion, especially when adjacent to the coastline and irrigated agricultural lands in the Mediterranean Region [43]. The introduction of *S. elaeagnifolium* propagules in natural land-use types is probably associated with the numerous agricultural areas adjacent or found within the borders of protected areas of the Greek Natura 2000 network [48,49].

## 4. Materials and Methods

### 4.1. Study Area, Sampling Strategy and Data Collection

**Figure 6:**
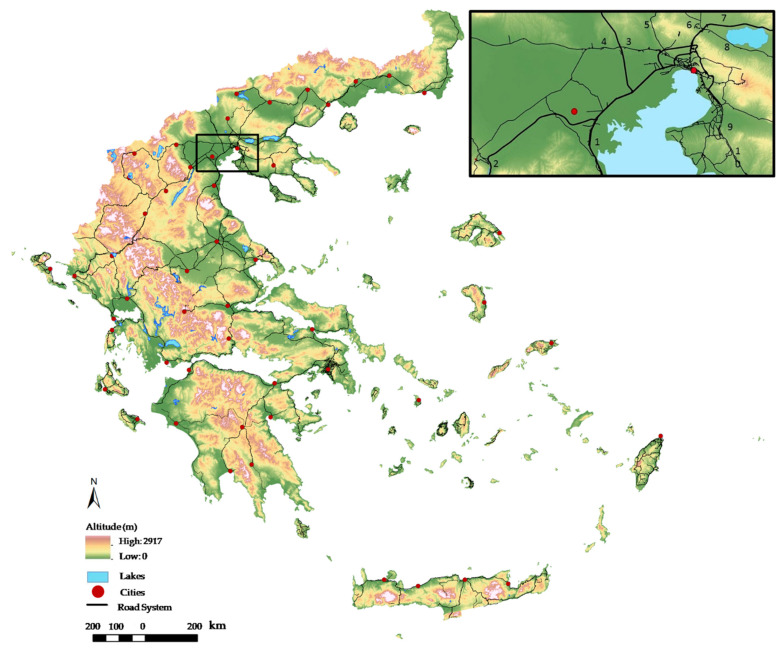
Geomorphology of the Greek territory with the basic road network connecting the capitals of local prefectures and overview of the inventories performed for *Solanum elaeagnifolium* (black lines). The zoomed-in area indicates an example of individual inventories performed from one city (Thessaloniki, Northern Greece) to other destinations such as exit roads to: Athens (1), Igoumenitsa (2), Edessa (3), Evzoni (4), Kilkis (5), Serres (6), Alexandroupoli (7), Kavala (8), northern Chalkidiki (9) and southern Chalkidiki (10).

First, to get an overview of the previously known distribution of *S. elaeagnifolium* in Greece, we compiled all known occurrences from the literature [14,19,20,21]. Then, in order to record the current *S. elaeagnifolioum* populations in Greece, we did a so-called drive-by survey (e.g., [50]). Therefore, we repeatedly traversed part of the national road network of Greece (Figure 6) during the period from 2000 to 2020 (see Supplementary S1). The sampling plan (Figure 6) covered the basic road network connecting all major Greek cities (capitals of local prefectures) with smaller satellite cities and other settlements, also transecting many rural landscapes and almost half of the protected areas of Greece. The altitudinal range of the surveyed terrain was 0–2200 m. In total, we surveyed 150 routes connecting 54 Greek cities. Most routes started in the center of a specific city and finished in the center of a settlement or another city (Figure 6). From the center of each city, we recorded per kilometer the distribution of *S. elaeagnifolium* populations along the basic exit roads leading to different destinations (Figure 6). This was made possible by using the car’s kilometer counter, which was manually set at zero before starting the inventory of each route from a specific city center to another city center. For routes between prefecture capitals, we also crosschecked the presences and absences per kilometer according to the kilometer road signs, which indicate the exact distance measured from a specific city center to another one along the Greek primary road network. The surveyed road network included multilane highways, the old interstate highway system and regional, local and rural roads. The total length of the surveyed road network was 15,736 km, corresponding to 21.54% of the total national network. The survey included all major national highways (922 km) and 4589 km (29%) of old interstate roads, 3977 km (25.3%) of rural roads and 6248 km (39.7%) of local roads (Table 2). The survey covered all administrative regions of Greece (except Mount Athos, which is an autonomous region on a peninsula with many monasteries in Northern Greece, and has restrictions on the movement of people and goods). Each of the selected 150 routes was surveyed at least 3–5 times during 2000–2020 (resulting in a total sampling effort of c. 100,000 km), and the presence/absence records were aggregated across the multiple surveys (see Supplementary S1).

**Figure 7:**
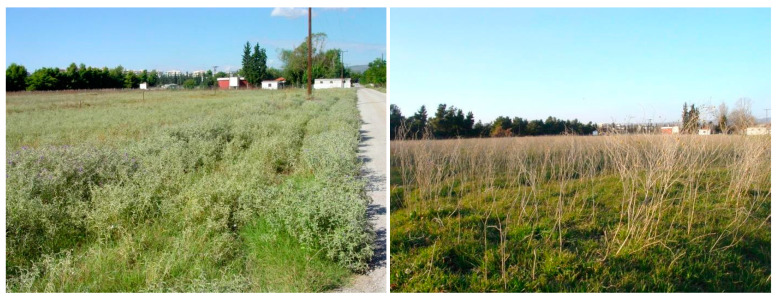
Cultivated fields in the Sindos area (Thessaloniki, Northern Greece) invaded by *Solanum elaeagnifolium* during summer (**left**) and winter (**right**).

**Figure 8:**
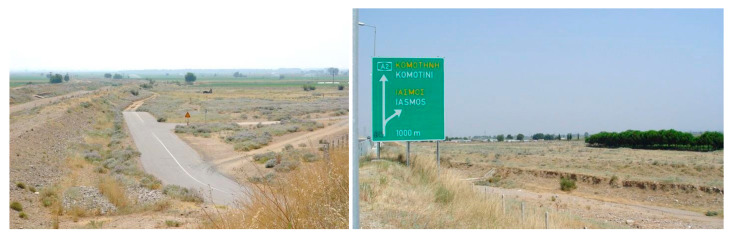
Rural roadsides next to multilane highways invaded by *Solanum elaeagnifolium* in the Iasmos area (northeastern Greece) during summer.

Observations of *S. elaeagnifolium* populations along the road verges were made by two scientists while driving in one car at a speed of 40–70 km/h. To avoid possible confusion with other species, all surveys were performed in summer and autumn, as in these seasons *S. elaeagnifolium* individuals are in full flower and/or fruiting, and consequently are evident from a distance due to their contrast with dried annual therophytes or other nonsummer flowering perennial plants (Figure 7 and Figure 8). Each time we encountered a population of the target species, we stopped the car for a while to record the geographical coordinates with portable GIS devices, and to record with binoculars the species’ presence in the surrounding area (a radius of c. 1 km).

### 4.2. Data-Set Compilation and Statistical Analysis

The presence and absence records of *S. elaeagnifolium* were entered into the GIS environment (version 10.1, ArcGIS^®^ software by ESRI, Redlands, CA, USA), and each record was assigned to a 1 km^2^ grid cell. More precisely, we created a grid (1 km × 1 km) on the examined road network, and on each grid cell, we recorded the absence or presence of *S. elaeagnifolium* based on the field observations. The presence and absence layer (hereafter distribution cells of *S. elaeagnifolium*) comprised 15,723 km^2^ in total (11.9% of the Greek territory). This was the basal layer to which data layers from selected geodatabases on environmental variables were added (Supplementary S2), following previously published methodology, i.e., point sampling and linking in a GIS environment over multiple polygon and raster layers extracted from selected thematic geodatabases with a resolution of 1 km^2^ [51,52].

To test for the effects of human-related factors [53], we included type and length of the roads, which provides a good proxy for human-intervention intensity in an area [17], and we also calculated the distance of each *S. elaeagnifolium* population from the center of settlements. Moreover, for each cell, we retrieved the altitudinal zones, soil properties and climate data, and we calculated the cover of the different land-use types (Corine Land Cover, 2000) (Supplementary S2).

For each of the 19 bioclimatic variables (dependent variables) (Supplementary S3, Table S2), we tested differences between the invaded areas (occupied grid cells) by *S. elaeagnifolium* and the noninvaded areas using univariate analysis of variance (ANOVA). As presented in the Results section, 1564 out of 15,736 cells were invaded by *S. elaeagnifolium*, which means that the noninvaded cells were approximately 10 times more numerous. Thus, to avoid any unbalanced comparison in ANOVA, we chose a subset of the noninvaded cells to obtain a similar number to the invaded ones. For making the subset, we used a random number generator that assigned a random number to each nonoccupied cell. Then the records having a value of this randomly assigned number below a certain value were selected for the subset until a number equal to the occupied cells was reached. This method was repeated to generate different subsets; we repeated our analysis 10 times to assure the robustness of our results. Prior to data analysis, we checked for assumptions of the method (i.e., for normality or symmetry). To adjust the *p* values for multiple testing, we applied Bonferroni corrections by dividing the standard 0.05 alpha by the number of tests.

A principal component analysis (PCA) on 19 bioclimatic variables was used to unveil how the occurrence of *S. elaeagnifolium* in Greece is related to climate. PCA axis 1 and 2 scores were also tested for differences between the invaded areas and the noninvaded areas following the methodology described above.

We performed chi-square tests to assess whether the distribution of elevation, soil parameters and land-use types differed among the invaded and noninvaded cells. All statistical analyses were performed with the SPSS v. 25 software package.

## 5. Conclusions

The invasive populations of *S. elaeagnifolium* are equipped to outcompete many native species (ruderals) of the Mediterranean region. Overall, the study herein shows that the climate, soil type and elevations of Greece provide limited restrictions to the occurrence of *S. elaeagnifolium*, while soil disturbance favors and road networks (serving both as corridors and sources of propagules) pave the way for the invasion of *Solanum elaeagnifolium*. Given that this alien invasive species has alarmingly increased its population in Greece within only a few decades, it is most likely that it will continue to swiftly invade new territories in Greece, the rest of the Mediterranean Region and worldwide.

Our study indicates that the priority areas in which preventative and control measures should be taken are lowlands, agricultural lands and road verges with disturbed and depleted soils. Unfortunately, these are quite commonplace in Mediterranean countries. Therefore, local, regional and global complex management regimes must be designed carefully, and they should be considered urgently and implemented promptly. Monitoring, prevention and immediate eradication measures are strongly advised as a remedy against the establishment in new areas. Possible absence of alertness on this “*Lernaean Hydra*”-like invasive plant should be avoided, especially by countries not suffering yet from its invasion.

## Figures and Tables

**Table 1:** Distribution of noninvaded and invaded areas by *Solanum elaeagnifolium* in Greece (percentage of cells) according to altitude classes.

Altitude Class (m)	Invaded Areas (% of Cells)	Non-Invaded Areas (% of Cells)
0–100	60.09	32.84
101–200	24.58	19.46
201–300	8.53	12.48
301–400	2.96	8.49
401–500	1.28	6.36
501–600	0.97	5.24
601–700	0.51	4.53
701–800	0.41	3.04
801–900	0.31	2.34
901–1000	0.15	1.70
1001–1100	0.10	1.13
1101–1200	0.10	0.76
1201–2200	-	1.62

**Table 2:** Length of different road types surveyed in Greece for noninvaded and invaded areas by *Solanum elaeagnifolium*.

Road Type	Road Length (km) with Occurrences (% of the Total Invaded)	Surveyed Length (km) (% of the Total Surveyed)	Total Length (km) in Greece (% of the Extant Network)
Multilane highways	674 (12.79)	922 (5.86)	2789 (3.82)
Old interstate roads	1276 (24.21)	4589 (29.16)	7316 (10.02)
Rural roads	1922 (36.47)	3977 (25.27)	19,003 (26.02)
Local roads	1399 (26.53)	6248 (39.71)	43,921 (60.14)
Total	5270 (100)	15736 (100)	73029 (100)

## Data Availability

Data are available on request.

## Supplementary Materials

The following are available online at https://www.mdpi.com/article/10.3390/plants10040805/s1, Supplementary S1: Partial inventories of *Solanum elaeagnifolium* conducted in different prefectures of Greece during the period 2000–2015, and their verification year confirming its current status of invasion (2016–2020); Supplementary S2: Selected geodatabases linked with the distribution (grid cells) of *Solanum elaeagnifolium* in Greece; Supplementary S3: Profile of nonbiotic parameters for the noninvaded and invaded areas by *Solanum elaeagnifolium* in Greece after linking multiple raster layers extracted from selected geodatabases in a GIS environment. For methodology used, see [52,53].
