# Supplementary material for: Investigating the Invasion Pattern of the Alien Plant Solanum elaeagnifolium Cav. (Silverleaf Nightshade): Environmental and Human-Induced Drivers
Source: Plants (Basel). 2021 Apr 20;10(4):805. doi: 10.3390/plants10040805 (PMC8072529; doi:10.3390/plants10040805)
Supplement: Supplementary file 1 [file plants-10-00805-s001.zip › Round 3-Appendix S1.pdf]

Supplementary material

Appendix S1: Partial inventories of *Solanum elaeagnifolium* conducted in different prefectures of Greece during the period 2000-2015, and their verification year confirming its current status of invasion (2016-2020)

Greek prefecture	Partial inventories / years	Verification year	Total inventories
Eastern Macedonia-Thraki	3 / 2007, 2008, 2009	2016	4
Central Macedonia	4 / 2000, 2005, 2010, 2015	2020	5
Western Macedonia	3 / 2008, 2015, 2016	2020	4
Epirus	4 / 2001, 2006, 2010, 2014	2018	5
Thessaly	4 / 2007, 2010, 2013, 2015	2020	5
North Aegean Islands	3 / 2008, 2012, 2013	2018	4
South Aegean Islands	4 / 2010, 2011, 2013, 2015	2017	5
Central Greece	5 / 2001, 2006, 2010, 2015	2019	5
Western Greece	3 / 2000, 2004, 2008	2017	4
Peloponnese	4 / 2001, 2002, 2010, 2011	2020	5
Ionian Islands	5 / 2007, 2008, 2009, 2010	2019	5
Crete	5 / 2010, 2013, 2014, 2015	2020	5
Attica	4 / 2003, 2007, 2010, 2015	2019	5
Agion Oros	3 / 2003, 2008, 2015	2017	4
