# Supplementary material for: Investigating the Invasion Pattern of the Alien Plant Solanum elaeagnifolium Cav. (Silverleaf Nightshade): Environmental and Human-Induced Drivers
Source: Plants (Basel). 2021 Apr 20;10(4):805. doi: 10.3390/plants10040805 (PMC8072529; doi:10.3390/plants10040805)
Supplement: Supplementary file 1 [file plants-10-00805-s001.zip › Round 3-Appendix S2.pdf]

Supplementary material

Appendix S1. Selected geodatabases linked with the distribution cells of *Solanum elaeagnifolium* in Greece.

1. AGGLOMERATIONS: Agglomeration data derived from the Greek Institute for the Management of Information Systems (http://geodata.gov.gr/).
2. ROAD NETWORK: The road network in Greece as appearing in OpenStreetMap contributors (Geofabrik GmbH and OpenStreetMap Contributors, http://download.geofabrik.de), (Neis & Zipf, 2012).
3. CLIMATE DATA: WorldClim dataset (mean values of 30 years) (Hijmans et al., 2005, http://www.worldclim.org/).
Bioclimatic variables: Annual Mean Temperature, Mean Diurnal Range, Isothermality, Temperature Seasonality, Max Temperature of Warmest Month, Min Temperature of Coldest Month, Temperature Annual Range, Mean Temperature of Wettest Quarter, Mean Temperature of Driest Quarter, Mean Temperature of Warmest Quarter, Mean Temperature of Coldest Quarter, Annual Precipitation, Precipitation of Wettest Month, Precipitation of Driest Month, Precipitation Seasonality, Precipitation of Wettest Quarter, Precipitation of Driest Quarter, Precipitation of Warmest Quarter, Precipitation of Coldest Quarter
4. LAND COVER: CORINE Land Cover 2000 database (EEA, 1995; Copernicus Land Monitoring Services, 2015, http://www.eea.europa.eu/publications/COR0-landcover, data as of 2012).
5. TOPOGRAPHY: Elevation map of Greece (contour lines 100 m) derived from the Greek Institute for the Management of Information Systems (http://geodata.gov.gr/).
6. SOIL PROPERTIES: European Soil database (ESDB v2.0) from the European Commission – DG JRC (2004).
7. ADMINISTRATIVE REGIONS OF GREECE (NUTS 2): Data from the Greek Institute for the Management of Information Systems (http://geodata.gov.gr/).

References

Copernicus Land Monitoring Services. CORINE Land Cover. European Environmental Agency, Copenhagen, Denmark, 2015. Available at: http://land.copernicus.eu/pan-european/corine-land-cover (accessed 1/9/202020).

European Commission – DG JRC. The European Soil Database distribution version 2.0, European Commission and the European Soil Bureau Network, CD-ROM, EUR 19945 EN, 2004. Available at: http://esdac.jrc.ec.europa.eu/content/european-soil-database-v20vector-and-attribute-data (accessed 1/9/2020).

Hijmans, R.J., Cameron, S.E., Parra, J.L., Jones, P.G., Jarvis A. Very high resolution interpolated climate surfaces for global land areas. *Intern. J. Climatol.* **2005**, 25, 1965–1978.

Neis, P., Zipf. A. Analyzing the contributor activity of a volunteered geographic information project: The case of OpenStreetMap. ISPRS Intern. *J. Geo-Inform.* **2012**, 1, 146-165.
