# Supplementary material for: Investigating the Invasion Pattern of the Alien Plant Solanum elaeagnifolium Cav. (Silverleaf Nightshade): Environmental and Human-Induced Drivers
Source: Plants (Basel). 2021 Apr 20;10(4):805. doi: 10.3390/plants10040805 (PMC8072529; doi:10.3390/plants10040805)
Supplement: Supplementary file 1 [file plants-10-00805-s001.zip › Round 3-Appendix S3.pdf]

Supplementary material

Appendix S3. Profile of non-biotic parameters for the non-invaded and the invaded areas by *Solanum elaeagnifolium* in Greece after linking in a GIS environment multiple raster layers extracted from selected geodatabases (for methodology used see Krigas et al. 2010, 2012).

Table S1. Main soil properties and related most significant categories as retrieved from the European Soil Database (ESDB) v2.0* for the invaded and the non-invaded areas by *Solanum elaeagnifolium* in Greece with results of chi-square test for soil properties.

Soil properties	Category	Percentage of records (%) of invaded areas (Percentage % in respect to the total areas surveyed)	Percentage of records (%) of non-invaded areas (Percentage % in respect to the total areas surveyed)	Percentage (%) of invaded areas / Percentage (%) of non-invaded areas
**Parent material** *Pearson Chi-Square: Value 109.271, Asymptotic Significance (2-sided) <0.0001*	fluvial clays, silts and loams	46.81 (8.10)	44.95 (91.90)	1.04
	limestone	16.69 (5.60)	23.94 (94.40)	0.70
	acid regional metamorphic rocks	12.77 (8.10)	12.14 (91.90)	1.05
**FAO soil type** *Pearson Chi-Square: Value 138.60, Asymptotic Significance (2-sided) <0.0001*	Calcaric Fluvisol	20.29 (8.50)	18.36 (91.50)	1.11
	Calcaric Regosol	14.48 (6.60)	17.35 (93.40)	0.83
	Calcaric Lithosol	13.58 (5.30)	20.37 (94.70)	0.67
	Chromic Luvisol	11.46 (9.40)	9.31 (90.60)	1.23
	Eutric Cambisol	6.14 (18.00)	6.96 (82.00)	0.88
	Eutric Lithosol	4.58 (8.80)	6.44 (91.20)	0.71
	Vertic Cambisol	6.38 (6.90)	5.58 (93.10)	1.14
	Calcaro-Chromic Vertisol	7.94 (5.70)	3.05 (94.30)	2.60
**Texture** *Pearson Chi-Square: Value 90.367, Asymptotic Significance (2-sided) <0.0001*	Fine (35% < clay < 60%)	63.83 (7.10)	67.29 (92.90)	0.95
	Coarse (18% < clay > 65% sand)	15.63 (7.80)	15.11 (92.80)	1.03
	Medium (18% < clay < 35% and >= 15% sand)	9.57 (18.00)	9.38 (82.00)	1.02
**Subsoil easily available water capacity** *Pearson Chi-Square: Value 66.786 Asymptotic Significance (2-sided) <0.0001*	M = Medium (100 - 140 mm/m)	44.84 (9.40)	36.44 (90.60)	1.23
	H = High (140 - 190 mm/m)	23.32 (6.80)	27.12 (93.20)	0.86
	VL = Very low (~ 0 mm/m)	20.54 (5.60)	29.12 (94.40)	0.71
**Depth to rock** *Pearson Chi-Square: Value 51.55, Asymptotic Significance*	D = Deep (80 - 120 cm)	33.06 (9.20)	27.61 (90.80)	1.20
	S = Shallow (< 40 cm)	21.52 (5.70)	29.88 (94.30)	0.72
	M = Moderate (40 - 80 cm)	20.54 (7.50)	21.40 (92.50)	0.96

(2-sided) <0.0001	V = Very deep (> 120 cm)	20.30 (8.50)	18.48 (91.50)	1.10
**Base saturation of the topsoil** *Pearson Chi-Square: Value 14.07, Asymptotic Significance (2-sided)< 0.001*	High (>75 %)	61.05 (7.60)	62.31 (92.40)	0.98
**Topsoil organic carbon content** *Pearson Chi-Square: Value 5.772, Asymptotic Significance (2-sided)=0.016*	L = Low (1 - 2 %)	72.09 (8.30)	67.45 (91.70)	1.07
	V = Very low (< 1 %)	23.32 (6.20)	29.92 (93.80)	0.78

*Composed of the Soil Geographical Database of Eurasia at a scale of 1:1000000 (version 4 beta) and the Pedotransfer Rules Database (v2.0), with raster resolution of 1 km^2 and FAO soil types (CORINE soil classification).

Table S2. Mean values of temperature and precipitation bioclimatic variables for the invaded and non-invaded areas by *S. elaeagnifolium* and results of GLM models testing differences on equal numbers of confirmed presences and randomly generated true absences. ***: *P*<0.001 (*P* values have been adjusted according to Bonferroni correction). NS: Non Significant. Data from the WorldClim database (Guarino et al., 2002; Hijmans et al., 2005) with a raster resolution of 1km^2 (average values of 30 years).

**Variable**	**Mean values ± STDEV in invaded areas (n = 1564)**	**Mean values ± STDEV in non-invaded areas (n = 14172)**	***F***	***P***
Annual Mean Temperature (°C)	15.76 ±1.71	15.97 ±2.16	8.50	NS
Temperature Annual Range (BIO5-BIO6) (°C)	28.62 ±3.45	26.63 ±4.29	105.72	***
Temperature Seasonality (STDEV *100)	670.14 ±65.44	623.53 ±79.2	172.60	***
Mean Diurnal Range {Mean of monthly (max temp - min temp)}	9.61 ±1.47	9.05 ±1.85	42.11	***
Isothermality { Mean Diurnal Range/ Temperature Annual Range) (* 100)}	3.3 ±0.19	3.32 ±0.27	11.40	NS
Max Temperature of Warmest Month (°C)	31.46 ±1.91	30.62 ±2.22	59.00	***
Min Temperature of Coldest Month (°C)	2.85 ±2.8	3.99 ±3.49	59.03	***
Mean Temperature of Wettest Quarter (°C)	9.82 ±2.33	10.07 ±2.5	6.48	NS
Mean Temperature of Driest Quarter (°C)	24.22 ±1.62	23.93 ±1.95	6.60	NS
Mean Temperature of Warmest Quarter (°C)	24.47 ±1.6	24.07 ±1.93	15.00	NS
Mean Temperature of Coldest Quarter (°C)	7.41 ±2.33	8.26 ±2.9	49.00	***
Annual Precipitation (mm)	56.49 ±16.38	66.54 ±20.8	105.59	***

Precipitation Seasonality (Coef. of Var.) (mm)	4.81 ±2.03	6.07 ±2.27	142.40	***
Precipitation of Wettest Month (mm)	8.8 ±3.88	11.59 ±4.77	154.08	***
Precipitation of Driest Month (mm)	1.34 ±0.74	1.06 ±0.87	56.54	***
Precipitation of Wettest Quarter (mm)	23.67 ±10.08	30.88 ±12.45	154.16	***
Precipitation of Driest Quarter (mm)	5.39 ±2.52	4.3 ±3.08	68.43	***
Precipitation of Warmest Quarter (mm)	5.8 ±2.74	4.68 ±3.22	60.74	***
Precipitation of Coldest Quarter (mm)	21.22 ±9.22	27.48 ±11.08	143.89	***

References

Guarino, L., Jarvis, A., Hijmans, R.J., Maxted, N. Geographic information systems (GIS) and the conservation and use of plant genetic resources. In Managing plant genetic diversity, Engels, J.M.M., Ramanatha Rao, V., Brown, A.H.D., Jackson, M.T. (Eds); International Plant Genetic Resources Institute: Rome, Italy, 2002; pp. 387–404.

Hijmans, R.J., Cameron, S.E., Parra, J.L., Jones, P.G., Jarvis, A. 2005. Very high-resolution interpolated climate surfaces for global land areas. *Intern. J. Climatol.* **2002**, 25, 1965–1978.

Krigas, N., Mouflis, G., Grigoriadou, K., Maloupa, E. Conservation of important plants from the Ionian Islands at the Balkan Botanic Garden of Kroussia, N Greece: using GIS to link the in situ collection data with plant propagation and ex situ cultivation. *Biodivers. Conserv.* **2010**, 19, 3583–3603. DOI:10.1007/s10531-010-9917-7.

Krigas, N., Papadimitriou, K., Mazaris, A.D. GIS and ex situ plant conservation. In Application of Geographic Information Systems, Alam, B.M., Ed.; InTechopen.com: Rjieka, Croatia, 2012; pp. 153-174.
